# Investigating microplastics and potentially toxic elements contamination in canned Tuna, Salmon, and Sardine fishes from Taif markets, KSA

**DOI:** 10.1515/biol-2021-0086

**Published:** 2021-08-23

**Authors:** Nahed Ahmed Hussien, Amaal Mohammadein, Ehab M. Tantawy, Yassir Khattab, Jamila S. Al Malki

**Affiliations:** Department of Biology, College of Science, Taif University, P.O. Box 11099, Taif 21944, Saudi Arabia; Research and Development Sector, EGYVAC, VACSERA, Giza 12311, Egypt

**Keywords:** microplastics, toxic elements, canned fish, Tuna, Salmon, Sardine, Taif, KSA

## Abstract

Microplastics (MPs) have been documented in different foodstuffs and beverages, that could affect human health due to their ingestion. Furthermore, seafood contamination with MPs puts pillars of food availability and utilization at risk. The present study investigates MPs and toxic elements pollution in commercially canned fishes from Taif governorate markets. Seven different canned fishes’ brands were used in the present study from different manufacturer countries and purchased from Taif markets. Tissue samples were digested by 10% of KOH; then, dry filters were analyzed by Fourier-transform infrared spectroscopy to detect MPs. Filtrates were used to detect any potentially toxic elements by inductively coupled plasma. Different MPs were detected in edible tissue, such as canned Tuna contaminated with nylon, 1,2-polybutadiene, and ethylene vinyl alcohol. Sardines contain ethylene vinyl alcohol and poly(vinyl stearate), but Salmon does not have any MPs. Different elements were present in the selected samples in the decreasing order of Al > Se > Zn and traces of As and Sb. Canned fishes were contaminated with MPs and potentially toxic elements. This contamination could be a warning of the potential health risks with the long-term exposure. Therefore, it is recommended to include micro-, meso-, and even nanoplastics in the guidelines of testing food safety management systems.

## 1 Introduction

Fish represent a good source of unsaturated fatty acids (including Omega-3), fat-soluble vitamins, proteins (including essential amino acids), and different elements (calcium, fluorine, iodine, and phosphorus) [1]. Therefore, fish and other seafood are considered as healthy and balanced meals for humans [2]. Canned fish is widely consumed in various countries of different continents, including the Kingdom of Saudi Arabia (KSA), Libya, Turkey, Iran, USA, and Portugal [3]. It was reported that Tuna, Salmon, and Sardine are the most consumed canned fishes in KSA [4].

Fishes are constantly exposed to different pollutants in contaminated waters. However, fish represents the final link in the aquatic food chain; therefore, detecting any contaminant in fish tissue must be related to marine environmental pollution [5].

Plastics have been found everywhere in terrestrial and aquatic ecosystems, that has increased rapidly worldwide. It was assessed that about 4.8–12.7 million metric tons of plastics entered the oceans by 2010 [6]. Large disposable plastics in the aquatic ecosystem were subjected to continuous degradation (mechanical, chemical, and photolytic), leading to smaller particles such as mesoplastics, microplastics (MPs), and nanoplastics [7,8,9,10].

Recently, different studies have reported the presence of micro- (0.001–1 mm) and mesoplastics (1–10 mm) in the alimentary canal of a few fish species [11,12,13]. Ingested micro/mesoplastics could be mistaken for food or could be found in other pelagic and benthic marine biotas as a meal for other fishes [14]. However, few studies report MPs’ ability to translocate from the digestive canal to other organs [15], leading to fish toxicity [8,16,17]. In our peer knowledge, rare research studies are focused on micro/mesoplastic estimation in edible fish tissues [18]. Their potential risks on biotic fauna or humans have not been well studied until now. However, MPs accumulation in aquatic biota, especially edible tissues, may put the health of seafood consumers at risk due to hazardous compounds [9,19]. During the last two decades, MPs have been considered an ecotoxicological risk due to their physical damage, oxidative stress, genotoxicity, growth inhibition, metabolism disorders, and liver metastasis [20,21,22,23].

Canned fish as a processed seafood product is directly consumed without any further cleaning process, and there is no information on micro/mesoplastic loads found in this product. Therefore, it is essential to evaluate the presence of MPs in those products from a human health perspective. However, investigating plastic particulates in foodstuffs is not included in any end product’s quality and safety assessment as per the International Standardization Organization rules (ISO) [24].

MPs can be chemically inert, small-sized, and with large surface areas facilitating the adsorption of toxic materials [25,26], although they could enhance the bioaccumulation of other pollutants present in water, including organic [27] and inorganic (potentially toxic elements) pollutants [28,29,30].

The present study aims to estimate MPs and element contamination in commercially canned fishes (Tuna, Salmon, and Sardine), frequently consumed in KSA. Fourier-transform infrared spectroscopy (FTIR) and inductively Coupled Plasma-Mass Spectrometry (ICP-MS) were used to detect MPs and metallic elements contamination, respectively.

## 2 Materials and methods

### 2.1 Sample collection

**Figure 1:**
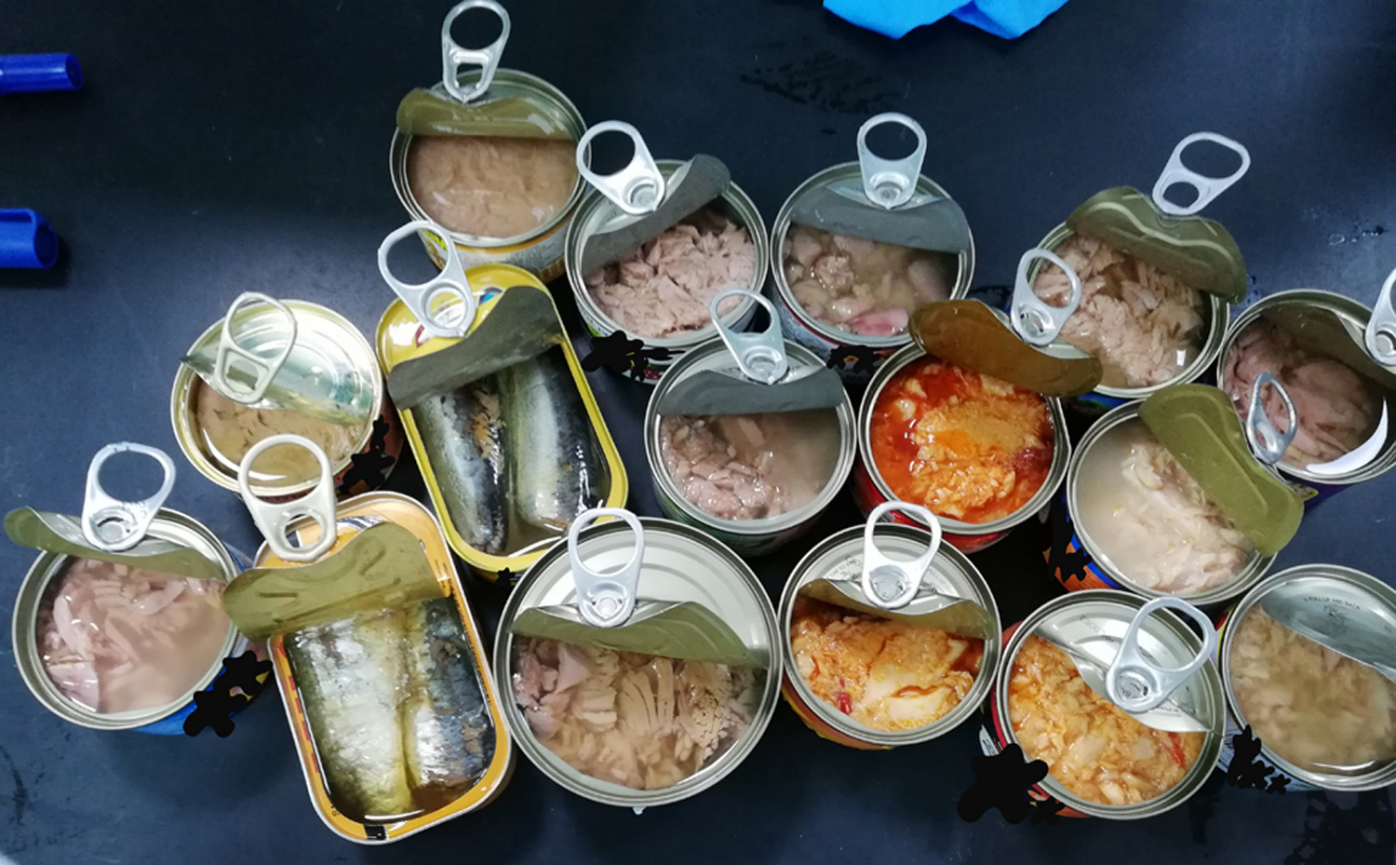
Different canned fishes that were used in the present study.

**Figure 2:**
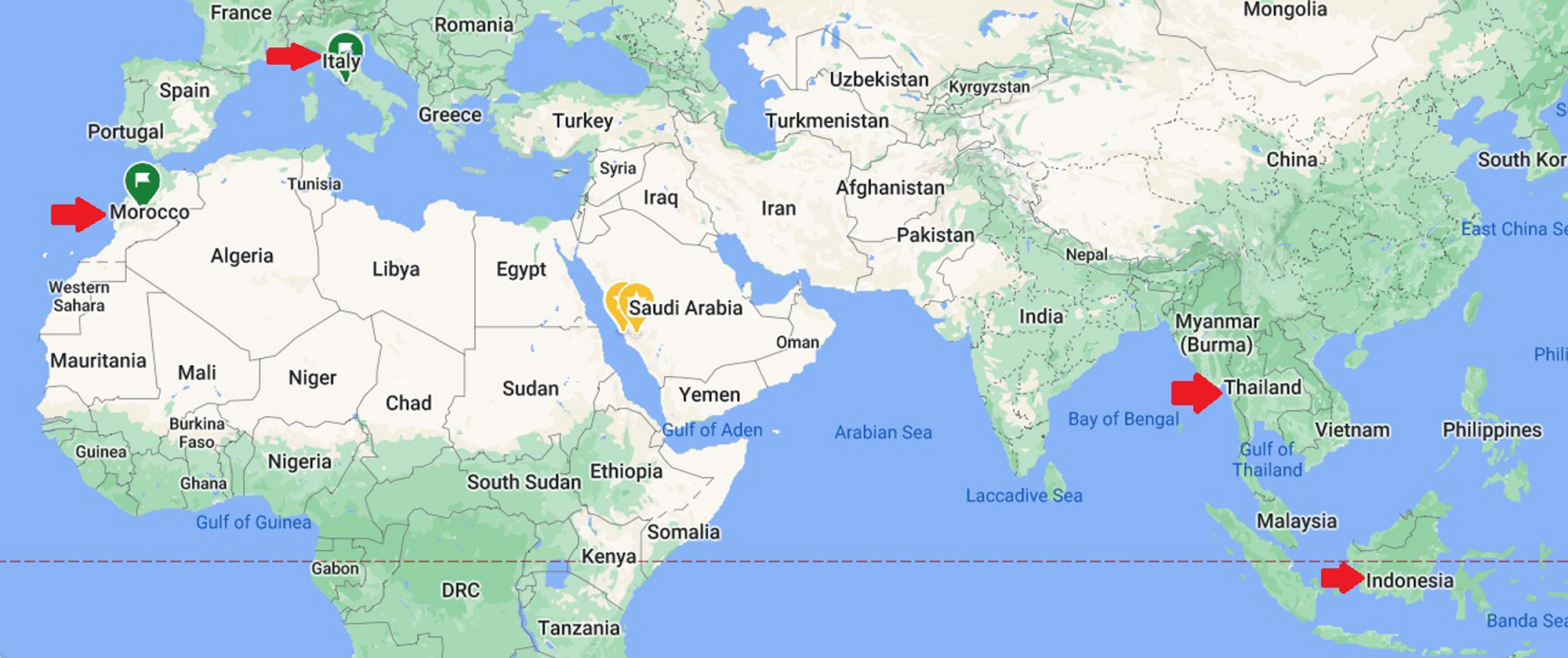
Map refers to the countries of selected canned fishes (Red arrows), according to google map 2020.

Seven different canned brands (triplicates) were used in this study: Canned Tuna, Salmon, and Sardine fishes were purchased from Taif governorate, KSA markets. They were manufactured in different countries: Tuna from Indonesia, Italy, and Thailand; Salmon from Indonesia and Thailand; Sardine from Indonesia and Morocco. Figure 1 shows the collected canned fish and Figure 2 refers to the countries of their manufacturer (map according to google map 2020). All data on the cans were recorded to know all the constituents found in them other than fish tissue (Table 1). It shows different concentrations of protein (g), total, saturated, and trans fats (g), sodium (mg), total carbohydrates (g), total sugar (g), cholesterol (mg), dietary fibers (g), and any other constituents (Nutrition facts/100 g) present in the cans. In addition, it refers to total weight (g), fish species, and manufacturer country.

**Table 1:** Data recorded on the collected cans used in the present study

N.o.	Fish	Country	Total weight (g)	Protein (g)	Total fat (g)	Saturated fat (g)	Trans fat (g)	Sodium (mg)	Total carbohydrate (g)	Total sugar (g)	Cholesterol (mg)	Dietary fiber (g)	Other constituents
1	Tuna	Indonesia	80–180	10–26.5	1.2–12.3	0.2–1.9	0–0.1	218–390	0	0	47–57	0	Water/vegetable oil, salt solution
2	Tuna	Italy	80	—	—	—	—	—	—	—	—	—	Olive oil, salt
3	Tuna	Thailand	90–100	17–26	0–18	0–6	0	156–360	0	0	46–50	0	Vegetable oil, salt solution
4	Salmon	Indonesia	94	14.5	1.3	0.4	0.1	315	0.5	0.5	42	0	Omega 3 (g) = 1.1
5	Salmon	Thailand	94	13.9	5.5	1.1	<0.1	448	2.2	1.6	—	—	Vegetable oil, chili, salt, sugar, natural colors
6	Sardine	Indonesia	250	—	—	—	—	—	—	—	—	—	Vegetable oil, chili, salt
7	Sardine	Morocco	250	22.3	14.5	2.8	5.5	240	0	0	0.035	0	Soya oil, salt, omega 3 (g) = 1.1

Dashes (—) refer to non-availability of data on the can. Nutrition facts/100 g.

### 2.2 Minimization of contamination with MPs

The working area was cleaned with ethanol before starting any treatment. Any plastic tools were prohibited from being used in this work. All glass tools were washed with autoclaved deionized water and then with ethanol before their use in this experiment. Any prepared solutions and incubated samples were kept in glasses and capped with aluminum foil to prevent plastic contamination. Gloves and cotton lab coats were worn during the experiment.

### 2.3 Sample preparation and MPs extraction

**Figure 3:**
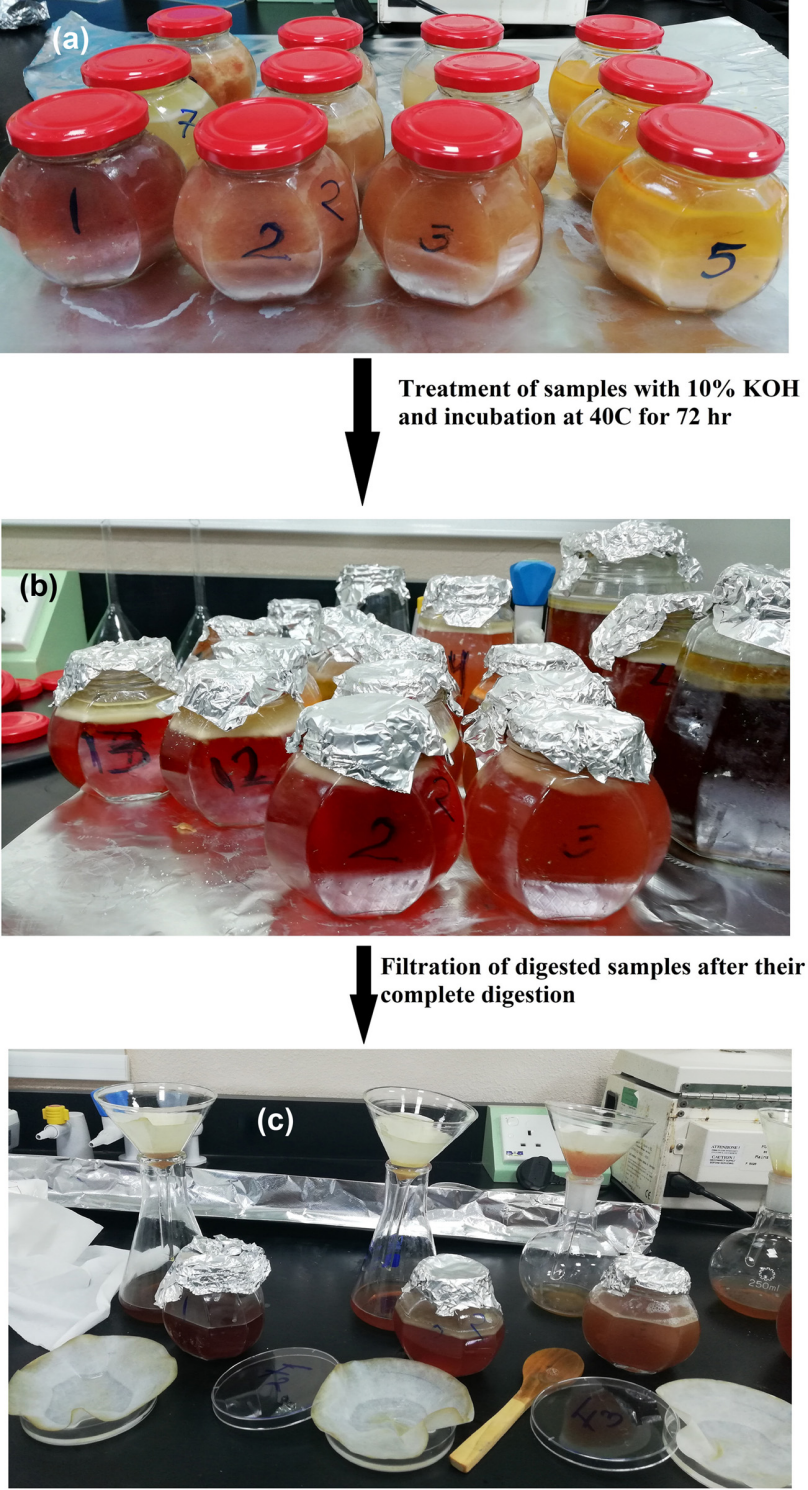
Steps of treatment: (a) treatment of fishes’ tissues with 10% of KOH and incubation for 72 h at 40°C, (b) the color of KOH becomes clear indicating complete digestion, and (c) filtration of the digested samples and filter papers soaked in Petri dishes.

Figure 3 shows treatment steps for MPs’ extraction in brief. About 40 g of canned fish (a separate can per brand) was used in treatment according to Rochman et al. [31] method with few modifications. Salt solution or oil found in the can with fish tissues were not discarded, and all were weighed and used together in evaluation. Samples were incubated in 10% of KOH (volume 3 times/weight) at 40°C for 72 h until complete tissue digestion. After complete digestion, NaI (4.4 M) was added to aid in MPs’ floating for its filtration. At the end of incubation time, the color of KOH turned from colorless to transparent red in all the samples except Sardine samples which appeared light brown in color, with successful digestion of the whole sample with no organic residues. Each sample was filtered separately through 8 μm cellulose filters (Whatman^®^ Grade 2). Then, filters were rinsed with autoclaved ultrapure water in Petri dishes, dried overnight at 60°C, and finally re-weighed to record MPs recovery weight per sample. Samples of the same brand were pooled and collected for further evaluation.

### 2.4 Microplastic visual identification

Collected dried filters of each sample were examined under a dissected microscope to help visualize MPs for further analysis by FTIR.

### 2.5 FTIR polymer identification

FTIR was used to characterize the collected particles. FTIR was extensively used for different particle identification, although it represents a fingerprinting technique. Organic and inorganic particles could be easily differentiated by yielding a unique spectrum by FTIR [32]. The FTIR spectra of samples were determined by an Agilent FTIR spectrometer at wavelength range 4,000–450 cm^−1^. OriginLab 2021 software was used to plot FTIR (absorbance to wavelength, cm^−1^). The spectra were aligned with the help of siMPle 2020 software (systematic identification of MPs in the environment, https://simple-plastics.eu/) against the reference spectra in its database to detect the type of particle.

### 2.6 Potentially toxic elements determination

Filtrates of all samples were used to detect different elements that could be accompanied by MPs contamination. Inductively Coupled Plasma-Mass Spectrometry (ICP-MS) was used for quantitative analysis of 20 different elements (Silver (Ag), Aluminum (Al), Arsenic (As), Barium (Ba), Beryllium (Be), Cadmium (Cd), Chromium (Cr), Cobalt (Co), Copper (Cu), Iron (Fe), Lead (Pb), Manganese (Mn), Molybdenum (Mo), Nickel (Ni), Antimony (Sb), Selenium (Se), Strontium (Sr), Titanium (Ti), Vanadium (V), and Zinc (Zn)) present in the samples.

### 2.7 Statistical analysis

Data were expressed as the mean value ± standard deviation (M ± SD). Statistical analysis was conducted to differentiate between all the groups and between the same canned fishes from different manufacturer countries using One-way ANOVA using GraphPad software (GraphPad, 2017)^®^. In which, *** indicates *P* ≤ 0.001, ** indicates *P* ≤ 0.01, * indicates *P* ≤ 0.05 and ns (non-significant) means *P* > 0.05.

## 3 Results and discussion

Different brands of canned fish from different manufacturer countries were used to determine any MPs and adsorbed elements pollution. Canned Tuna, Salmon, and Sardine are the most consumed seafood products found in KSA markets. All samples were digested entirely in 10% of KOH at the end of the treatment time and gave clear color, except for sardine samples because most of the fish body, including bones and skin, were found inside the can. It was reported that 10% of KOH is the most efficient, high-performing, low-cost digestion solution used in MPs extraction. The whole organic tissue was fully digested at 40°C in 48–72 h without negatively affecting the integrity of the plastic polymers. In addition, any other fragments present were successfully separated with the help of NaI to isolate MPs [8].

Dried filtered particles were examined under an inverted microscope (10×) for further examination. First, light colored particles and fibers with the same thickness were selected for FTIR examination to determine their type (data not shown). Then, FTIR was used to detect the chemical composition of the selected particles. The present work does not focus on MPs quantification but evaluates its presence and any accompanying pollutant. However, visual sorting is highly time-consuming, with a high error rate from 20% [33] to 70% [34] that increases with the decrease in the particle size.

Furthermore, it is hard even for an experienced person to distinguish between MP particles from chitin fragments, sand grains, diatom frustule fragments, or other non-polymers. NaI was used for MPs floatation, but non-polymer micro-debris could also float, leading to confusion and over-estimation. Randomly, ten different particles were selected from the filter of each brand sample, and their chemical composition was identified using FTIR.

**Figure 4:**
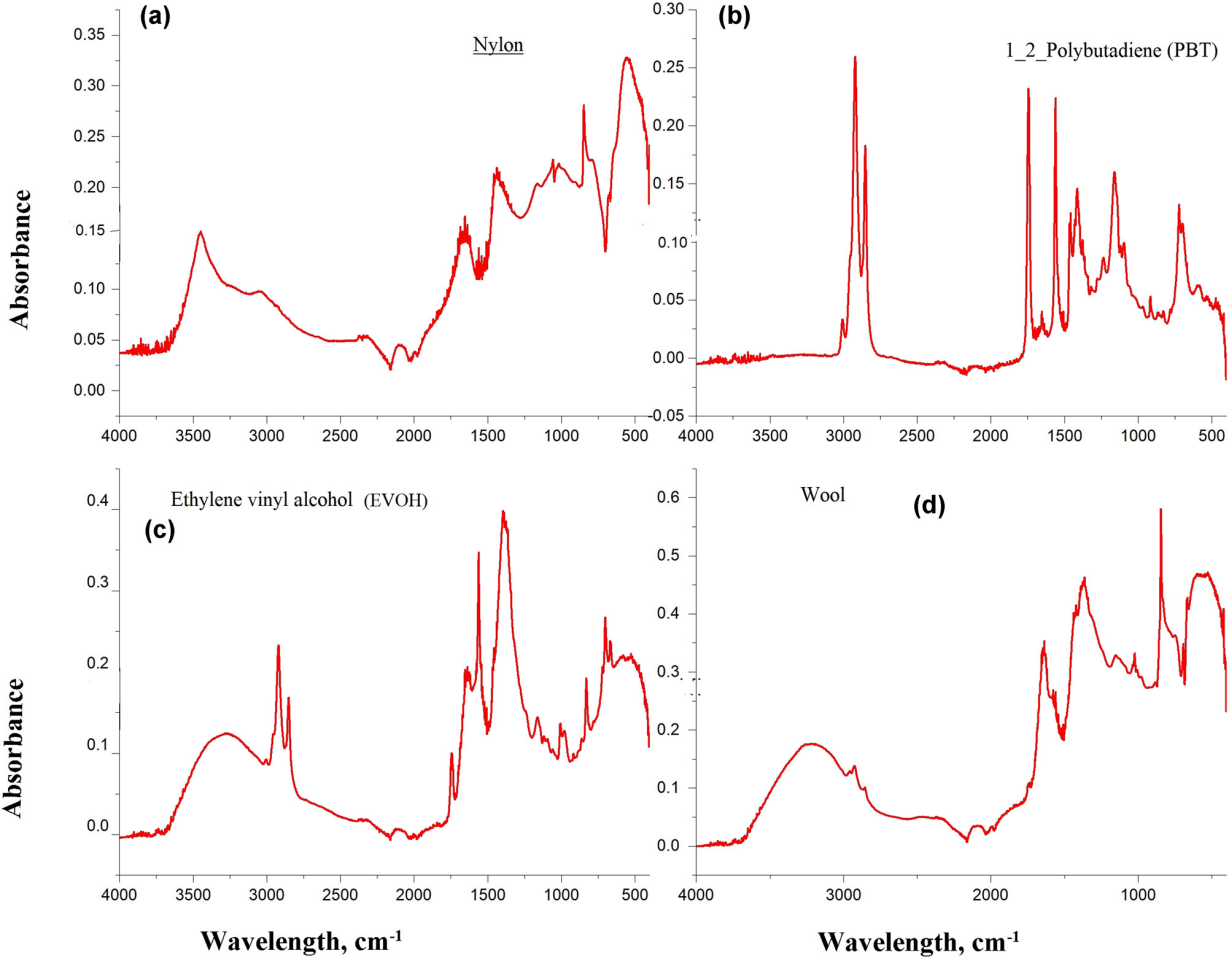
FTIR spectra of Nylon (a), 1,2-polybutadiene (PBT) (b), ethylene vinyl alcohol (EVOH) (c), and wool (d) that are present in samples.

**Figure 5:**
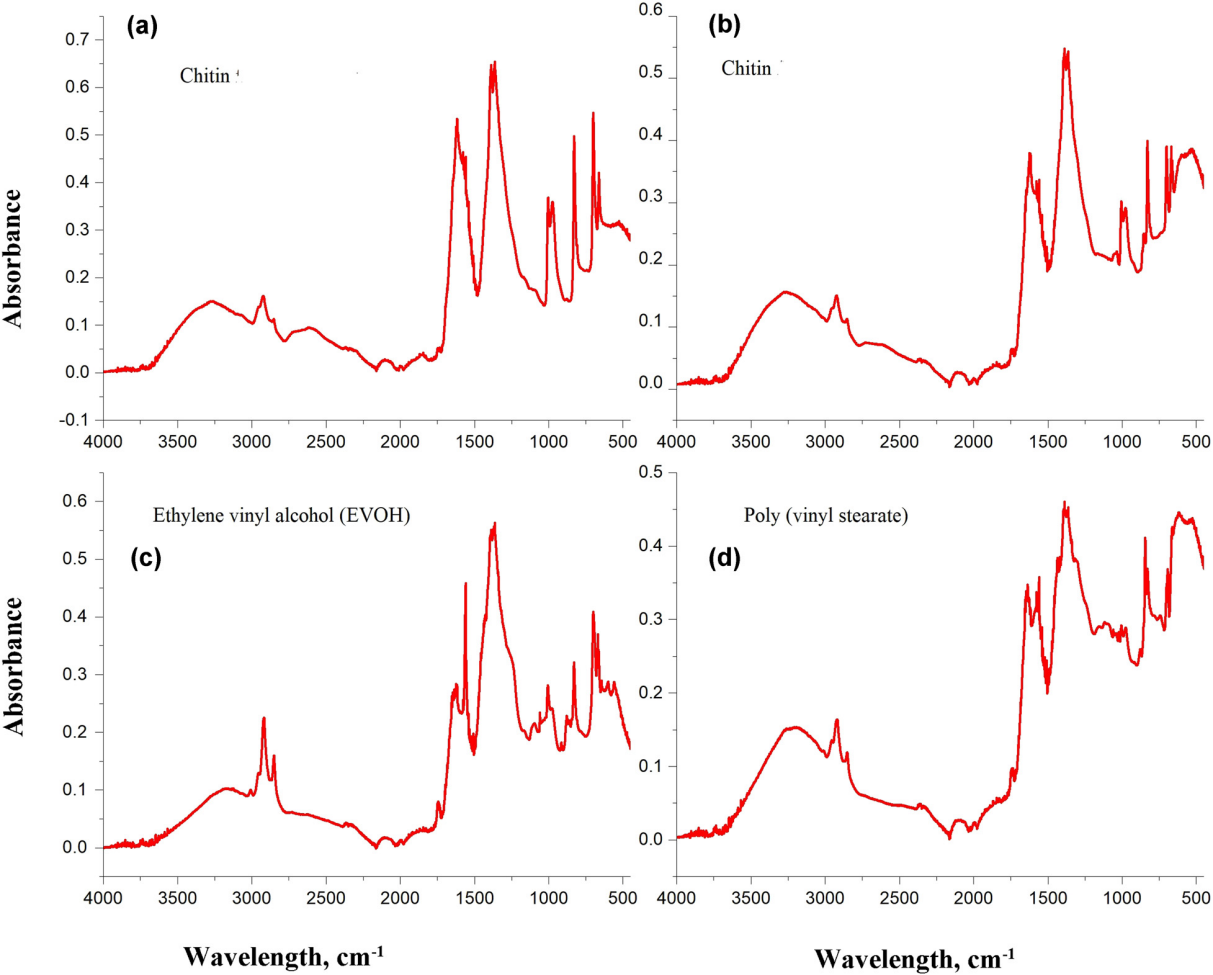
FTIR spectra of chitin protein (a and b), ethylene vinyl alcohol (EVOH) (c), and poly(vinyl stearate) (d) that are present in samples.

FTIR analyses of all selected particles were aligned by reference spectra in the database of siMPle 2020 software to report their type. Based on the report, most of the particles chosen from Tuna tissues from different manufacturer countries appeared to be synthetic polymers such as nylon, 1,2-polybutadiene (PBT), ethylene vinyl alcohol (EVOH, thermoplastics), and other natural polymers such as wool (Figure 4). All the selected particles from sardine filters are EVOH and poly(vinyl stearate) (Figure 5). However, none of the particles chosen from Salmon canned tissues from different manufacturer countries are MPs. As the spectra have shown, particles extracted from Salmon tissues consisted of chitin, as referred to in Figure 5. Few studies were found that reported MP particles in edible tissues of canned fishes. Canned Tuna and sardine tissues have MPs of different types, but Salmon tissue does not have any. The present result was consistent with Akhbarizadeh et al. [35]. They have recorded MP particles and fibers in canned tuna and mackerel fishes using light, fluorescence, micro-Raman microscopy, and scanning electron microscopy coupled with a dispersive energy X-ray. This could be due to different feeding habitats and the distribution of different species. It was reported that Tuna is a migratory fish and could concentrate large amounts of various pollutants [36], including MPs and heavy metals. In addition, Sardine fishes live in coastal waters [37] where different plastic particles (micro-, meso-, and macroplastics) are commonly dumped and can accumulate in their tissue [38,39]. In addition, Karami et al. [24] reported MPs, especially PP and PET, isolated from the canned Sardines and Sprats. They concluded that this contamination could be due to translocation of MPs into the edible tissues, improper gutting, or contamination from the canneries. In addition, contact materials during the cleaning and canning process and food additives could be possible sources of MPs contamination [35].

The present results were in agreement with Akhbarizadeh et al. [18], in which they reported the MPs bioaccumulation in gills and edible tissues of five different marine species: prawns (*Penaeus semisulcatus*), crabs (*Portunus armatus*), and fishes (*Epinephelus coioides*, *Platycephalus indicus*, and *Liza klunzingeri*). They have recorded different MP concentrations within different species, and it is found highly concentrated in gills more than the edible muscles. They concluded that motile, benthic, and deposit feeder species are more susceptible to deposited plastic particles on seabed sediments [40,41]. In addition, the MPs ingestion rate by aquatic biota depends on the MPs’ size, color, and organism’s size, vertical distribution, and feeding behavior [12,42,43,44,45]. Therefore, different hypotheses were expected regarding the passage of MPs to edible muscles that could transfer through the skin, gill, eye, blood circulation, and gut epithelium [9,21,47]. In addition, Collard et al. [46] suggested the passage of agglomerated MPs through microfold cells of the intestinal barrier and larger particles (110 μm) pass between cells in a paracellular manner [48].

**Figure 6:**
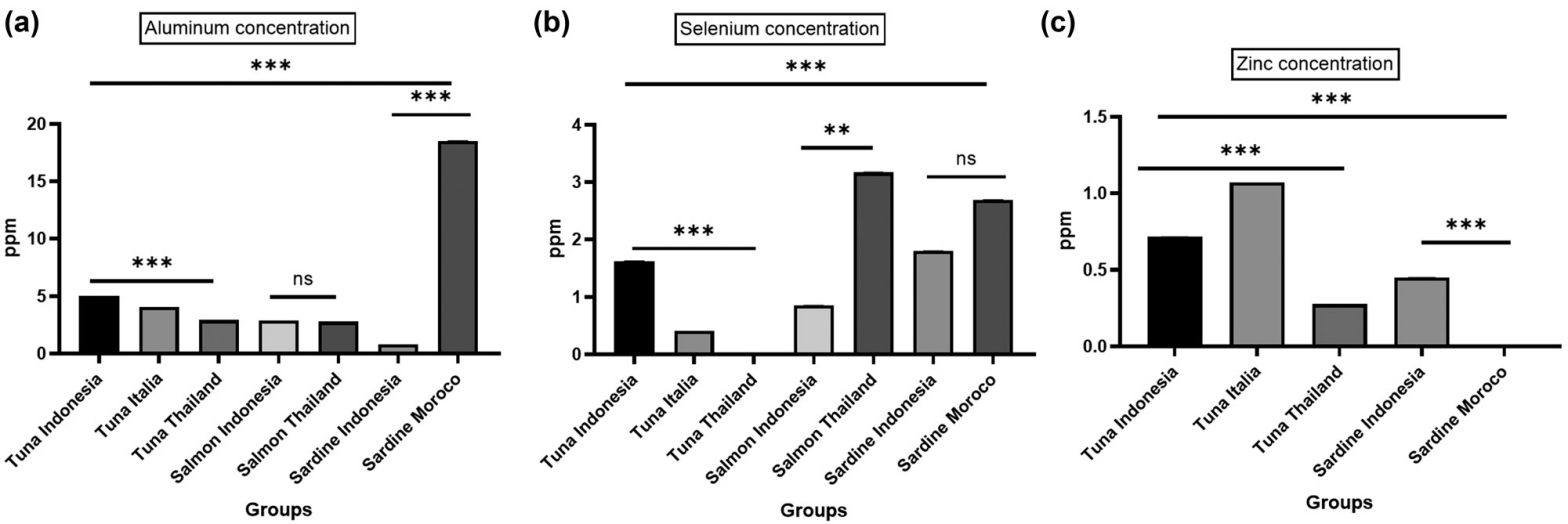
Assessment of different elements aluminum (a), selenium (b), and zinc (c) present in the filtrate of different canned fishes in ppm. In which, *** indicates *P* ≤ 0.001, ** indicates *P* ≤ 0.01, ns (non-significant) means *P* > 0.05 comparing related samples with each other.

Due to MPs’ small size and large surface area, different chemical pollutants in trace concentrations are easily adsorbed on their surfaces, including heavy metals, organochlorine pesticides, polychlorinated biphenyls, polycyclic aromatic hydrocarbons, and pharmaceuticals [30,49,50,51]. The filtrate was used to detect any trace toxic elements found in the canned fishes accompanied by MPs contamination. There are about fourteen different elements that are absent in the present study: Ba, Be, Cd, Cr, Co, Cu, Pb, Mn, Mo, Ni, Ag, Sr, Ti, and V. The highest Al concentration is significantly present in the Sardine Morocco (18.5701 ± 0.05) sample in comparison to other samples. Within the same fish species, Tuna Indonesia (5.0092 ± 0.001) significantly represents the highest Al concentration than other Tuna samples (other manufacturer countries). There is a non-significant difference between Salmon Indonesia and Thailand of Al concentration. However, Salmon Thailand significantly shows the highest Se concentration (3.1736 ± 0.004) in comparison to other groups’ samples. Within the same fish species, Tuna Indonesia (1.6269 ± 0.002) significantly represents the highest Se concentration than other Tuna groups. There is a non-significant difference between Se concentration for Sardine samples from Indonesia (1.8025 ± 0.001) and Morocco (2.6943 ± 0.002) (Table 2, Figure 6a and b).

**Table 2:** Concentration of different elements present in filtrate of different canned fishes in ppm

Element (ppm)	Tuna Indonesia	Tuna Italy	Tuna Thailand	Salmon Indonesia	Salmon Thailand	Sardine Indonesia	Sardine Morocco
Al	5.0092 ± 0.001	4.0514 ± 0.001	2.9435 ± 0.00	2.8861 ± 0.00	2.7924 ± 0.001	0.8268 ± 0.00	18.5701 ± 0.05
Sb	0.00	1.8476 ± 0.001	0.00	0.00	1.7192 ± 0.001	0.00	0.1277 ± 0.00
As	0.00	0.4087 ± 0.001	0.00	0.00	0.00	0.00	0.00
Se	1.6269 ± 0.002	0.4118 ± 0.001	0.00	0.8572 ± 0.002	3.1736 ± 0.004	1.8025 ± 0.001	2.6943 ± 0.002
Zn	0.7155 ± 0.001	1.0733 ± 0.003	0.2768 ± 0.001	0.00	0.00	0.4511 ± 0.001	0.00
Ba, Be, Cd, Cr, Co, Cu, Pb, Mn, Mo, Ni, Ag, Sr, Ti, V	0.00	0.00	0.00	0.00	0.00	0.00	0.00

The Tuna Italy group shows the highest significant concentration of Zn (1.0733 ± 0.003) in comparison to the other groups. Sardines from Indonesia show a low concentration of Zn (0.4511 ± 0.001), but the rest of the groups do not contain Zn (Table 2, Figure 6c). Tuna Italy is the only group that has a low concentration of As (0.4087 ± 0.001). In addition, Sb was found in three groups only: Tuna Italy (1.8476 ± 0.001), Salmon Thailand (1.7192 ± 0.001), and Sardine Morocco (0.1277 ± 0.00) (Table 2). It was known that metals are classified as essential (Cu, Zn, and Se), probably essential (Ni, V, and Co), and toxic (Al, As, Cd, Pd, and Hg) [52]. Although low concentrations of those elements are essential in seafood, high concentrations could be toxic [53,54]. It was reported that the metal concentrations in canned fish tissues were in the decreasing order of Al > Se > Zn, and only traces of As and Sb were detected in the selected sample groups from the Taif governorate market.

Different studies reported the presence of heavy metals in canned fishes, such as Hg, Zn, Cd, and others that could be within or out of the guidelines of the Food and Agriculture Organization (FAO)/World Health Organization (WHO). Ashraf et al. [4] reported different heavy metals in canned Salmon, Sardine, and Tuna fish muscles purchased in KSA (Zn = 3.80–23.9046 ppm, Pb = 0.03–1.97 ppm, and Cd = 0.01–0.69 ppm). According to the FAO, the present results estimate Zn concentration lower than the maximum permitted level of Zn in fish, which is 40–50 μg/g [55]. In agreement with the present results, Al Ghoul et al. [56] detected Zn, Al, and Sn in canned tuna fish commercialized in Lebanon within a limited range. In the present study, Al concentration in all the samples from different manufacturer countries (ranges from 0.8268 ± 0.00 to 5.0092 ± 0.001 ppm), except Sardine Morocco, are within the permissible limits for Al set by FAO/WHO as 60 mg/day. In agreement with previous studies, they record Al in canned tuna samples from Lebanon (Al = 4.756 μg/g), Indian (Al = 3.161 μg/g), and Canadian (1.806 μg/g) markets [5,56]. An increase in Al concentration in Sardine Morocco might be due to marine source Al contamination, leaching Al from the metal can, or from can coating liquid containing Al-based additives [57]. Salmon tissue has the highest essential Se element, low concentration of Al, a trace of Sb, the absence of other elements, and the lack of MPs contamination.

Generally, canned Salmon appears to be the safest processed seafood due to the absence of MPs contamination, toxic elements, and is rich in essential Se elements and Omega 3. However, microplastics were reported in canned Tuna, sardine and also as noted before in other foodstuffs, including canned Sprats, salt, and honey [9,24,58]. The long-term exposure to MPs could be a warning of the potential health risks. Annually, there is an increase in the plastic debris entering marine environments and constantly accumulates in the aquatic biota and seafood products. The accumulation of plastic particles was accompanied by other potentially toxic elements such as Al or others. Although, concerning the current load of MPs in canned Tuna and Sardines that might increase later, it is recommended to include micro-, meso-, and even nanoplastics in guidelines of testing food safety management systems.

## 4 Conclusion

In our peer knowledge, it is the first time to report MPs contamination in canned Tuna and Sardines from different manufacturers in KSA markets. MPs reported are nylon, PBT, EVOH, and poly(vinyl stearate). In addition, different elements were present in the selected samples in the decreasing order of Al > Se > Zn and only traces of As and Sb. However, Salmon tissue does not have any MPs and contains a low concentration of potentially toxic elements with higher concentration of essential Se. Therefore, microplastic pollution in processed seafood products that humans consume could be a potential risk to human health. According to the present results, we suggest the investigation and quantification of MPs to be included as one of the components of food safety management systems.
